# Correction: Influenza vaccination hesitancy in five countries of South America. Confidence, complacency and convenience as determinants of immunization rates

**DOI:** 10.1371/journal.pone.0308524

**Published:** 2024-08-01

**Authors:** Miguel Ángel González-Block, Emilio Gutiérrez-Calderón, Blanca Estela Pelcastre-Villafuerte, Juan Arroyo-Laguna, Yamila Comes, Pedro Crocco, Andréa Fachel-Leal, Laura Noboa, Daniela Riva-Knauth, Berenice Rodríguez-Zea, Mónica Ruoti, Elsa Sarti, Esteban Puentes-Rosas

There is an error in Table 5. The column labels were placed incorrectly.

**Table 5:** Odds ratio of influenza vaccination (at least once in the lifetime and in last year) and vaccine confidence, complacency and convenience, by risk group. All countries. Binary logistic multivariate regression analyses (enter method to select variables).

		OR	CI	p-value	OR	CI	p-value	OR	CI	p-value
Older adults	A B	1.635 1.709	1.429–1.871 1.501–1.944	< .001 < .001	1.662 1.268	1.372–2.012 1.076–1.494	< .001 < .005	1.247 1.220	1.112–1.398 1.095–1.358	< .001 < .001
Adults with risk factors	A B		1.183–1.452 1.229–1.507	< .001 < .001	1.715 1.414	1.448–2.031 1.213–1.648	< .001 < .001	1.289 1.337	1.168–1.422 1.211–1.475	< .001 < .001
Pregnant women	A B	1.562 1.437	1.368–1.784 1.270–1.625	< .001 < .001	1.272 1.093	1.070–1.513 0.938–1.273	< .001 NS	1.485 1.400	1.336–1.650 1.267–1.546	< .001 < .001
Mothers of children	A B	1.299 1.313	1.150–1.467 1.170–1.473	< .001 < .001	1.382 1.346	1.168–1.636 1.152–1.572	< .001 < .001	1.381 1.322	1.239–1.541 1.192–1.465	< .001 < .001

OR: Odds ratio; CI: Confidence Interval (95%). A: Vaccinated at least once in the lifetime; B: Vaccinated in the last year.
