# Supplementary material for: Influenza vaccination hesitancy in five countries of South America. Confidence, complacency and convenience as determinants of immunization rates
Source: PLoS One. 2020 Dec 11;15(12):e0243833. doi: 10.1371/journal.pone.0243833 (PMC7732123; doi:10.1371/journal.pone.0243833)
Supplement: S1 Questionnaire — (PDF) [file pone.0243833.s001.pdf]

Evisys

Survey on knowledge, attitudes and practices about the influenza vaccine
Group: adults with risk factors

City		Health establishment		Public<br>Private	1<br>2

Date	Day	Month	2018 Year	Start time	Hours : Minutes	Finishing time	Hours : Minutes	Questionnaire number	

Introduction

Good morning (afternoon), my name is _____ and I am a _____ surveyor for a study that seeks to find out how people like you feel about the flu vaccine. I would like to ask you a few questions related to this topic. You, like other people, have been randomly selected to represent the people who inhabit this city. I am going to ask you to answer a questionnaire that lasts approximately 15 minutes, about the influenza vaccine. The information you provide will be treated confidentially and anonymously, and will be used only for statistical purposes. This means that the personal data of the participants cannot be identified.

Instructions for coding responses:

Read the question and once the answer is obtained, circle the corresponding number in the "Code" column.

	QUESTION	ANSWER (Options)	Code	Pass to:
1	*Sex*	Male	1	
		Female	2	
2	*Age (years)*	Years		
3	*Civil status*	Single	1	
		Married	2	
		Free union	3	
		Divorced / Separated	4	
		Widow	5	
4	*Do you speak an indigenous language?*	Yes	1	
		No	2	
5	*Do you consider yourself indigenous*	Yes	1	
		No	2	
6	*What level of education do you have?*	Don't know how to read and write	1	
		Primary (complete or incomplete)	2	
		Secondary or equivalent (complete or incomplete)	3	
		Technical superior studies (complete or incomplete)	4	
		University studies (complete or incomplete)	5	
		Postgraduate (complete or incomplete)	6	
7	*Do you normally carry out any activity for which you receive monetary income?*	Yes	1	
		No, is a pensioner or retiree)	2	→ *9*
		No, is a student	3	→ *9*
		No, dedicated to the home	4	→ *9*

		No, has permanent limitations that impede working …...	5	→ 9
		No, is in a different situation to any of the above …..…………...…	6	→ 9
**8**	*From the following options, indicate the type of your main job*	Employee of a public institution	1	
		Employee of a private institution	2	
		Salaried worker	3	
		Own formal business (individual or family) ………… ...... ……	4	
		Independent consultant or professional ………………… .... ……	5	
		Informal employment or self-employment (individual or family) …..	6	
		Farmworker ……………………………………………….	7	
		Other..	8	
**9**	*Are you a member or beneficiary of any public social security institution? (Case of Peruvian institutions) (Only one answer option should be checked)*	Comprehensive Health Insurance (SIS) ……………………..… ..……	1	
		Social Security (Essalud) ……………………. ………..… .. …………	2	
		Health of the Armed Forces (FOSPEME, FOSMAR, ………..………	3	
		Health of the Peruvian National Police (SALUDPOL) ……………	4	
		None …………………………...……………………………..…..	5	
**10**	*Do you have any private health insurance, either as a holder or as beneficiary?*	Yes ……………………………………………..………….………	1	
		No ……………………………………………………….…………..	2	
		Knowledge of influenza		
**11**	*Do you know what influenza is?*	Yes ……………………………………………..………….………	1	
		No ………………………………………..……………….………..	2	→ 13
**12**	*Tell me some of the main symptoms of influenza*	Bed prostration …………………………………… ...….	Yes (1); No (2)	
		Diarrhea …..……………………………………………...…	Yes (1); No (2)	
	(Do not read the options, just wait for the answers and check the corresponding options	Vomit ……………………….…………………………..….	Yes (1); No (2)	
		High fever ……………………………………………… ..	Yes (1); No (2)	
		Shaking chills ……………………………………….....…	Yes (1); No (2)	
		Cough ………………………………..…………………...…	Yes (1); No (2)	
		Sore throat (pain when swallowing) …………… .. ……….…	Yes (1); No (2)	
		Stuffy nose (nasal congestion) …………………… ..… ..….	Yes (1); No (2)	
		Muscle and body aches (of joints)……………………………..…	Yes (1); No (2)	
		Headache ……………………..………………………….….	Yes (1); No (2)	
		Runny nose… .. …………………………………………… ..….	Yes (1); No (2)	
		Chest pain ……… ... ………. ……………………… . ……..….	Yes (1); No (2)	
		Stomach ache ..…..………….…. ……………………..….	Yes (1); No (2)	
		Fatigue (tiredness)… .. ……………………………………… ..….	Yes (1); No (2)	
		Irritability… .. ………………………………………… ..….	Yes (1); No (2)	
**13**	*Do you know that there is a flu vaccine?*	Yes ……………………………………………..………….………	1	
		No ………………………………………..…………………..…..	2	→ 23
**14**	*Regarding information about influenza, have you had access to it through:*	Triptychs or brochures …………………………………………	Yes (1); No (2)	
		Posters …………………………………………………………	Yes (1); No (2)	
		Radio ………………………………………………………..	Yes (1); No (2)	

		**Television ………………………………………………………..**	**Yes (1); No (2)**	
		**Health personnel …………..…………………………………..**	**Yes (1); No (2)**	
		**Other____** (Specify)	**Yes (1); No (2)**	
**15**	***Do you have information about:***	**when should you get the vaccine? …………………… ...……..**	**Yes (1); No (2)**	
		**to whom should the vaccine be given? …………… ... ……..…**	**Yes (1); No (2)**	
		**contraindications of its application? ……………. ………….……**	**Yes (1); No (2)**	
		**what are the possible effects?……………………….…………...**	**Yes (1); No (2)**	
**16**	***Do you think that the information you have about the influenza vaccine is sufficient?***	**Yes ………………………………………………..……………………**<br>**No ………………………………………..…………………………..**<br>**Don't know..………………………………………..…………………….**	**1**<br>**2**<br>**3**	
**17**	***<u>Where or from whom have you mainly obtained this information?</u>***	**Triptychs or brochures ………………………. ……………………**<br>**Posters ………………………………………. …… .. ………………**<br>**Radio ……………………………………………...………………..**<br>**Television ……………………………………………………………..**<br>**Health personnel …………..……………………………………………...**<br>**Relatives ………………………………………………..……………**<br>**Neighbors, friends, acquaintances …………………………. ………**<br>**Other: ____**<br>**(Specify)**	**1**<br>**2**<br>**3**<br>**4**<br>**5**<br>**6**<br>**7**<br>**8**	
ACCESSIBILITY AND AFFORDABILITY				
**18**	***Have you ever been recommended to get a flu shot?***	**Yes ………………………………………………..……………………...…**<br>**No ………………………………………..…………………………..**	**1**<br>**2**	 *→ 20*
**19**	***Who recommended it to you?***	**Health service center personnel (doctor, nurse, etc.)……………..…**<br>**Health campaign personnel (school, plaza, public places)………… ..**<br>**In my work ……………………..………………..……..…..……**<br>**Family members… .. ………………………. ………………… .. ………**<br>**Neighbors, friends, acquaintances…. ……. ………………… .. ……**<br>**Other: ____**<br>**(Specify)**	**1**<br>**2**<br>**3**<br>**4**<br>**5**<br>**6**	
**20**	***Do you know where to go to get a flu shot?***	**Yes ………………………………………………..……………………...…**<br>**No ………………………………………..…………………………..**	**1**<br>**2**	
**21**	***If you wanted to be vaccinated against influenza, do you think that you should be able to haveit applied in the health facilities in [Country]?***	**Yes ………………………………………………..………………………**<br>**No ………………………………………..…………………………..**<br>**Don't know..………………………………………..…………………….**	**1**<br>**2**<br>**3**	
**22**	***Is the vaccine available at the health facility where you go regularly?***	**Yes ………………………………………………..………………………**<br>**No ………………………………………..…………………………..**<br>**Don't know..………………………………………..…………………….**	**1**<br>**2**<br>**3**	
**23**	***How long does it take to get from your home to the***	**Hours** [ ] **Minutes** [ ]		

	health facility you go to regularly?			

ATTITUDES ABOUT INFLUENZA

24. Perceptions regarding influenza

QUESTION		L E V E L					
		Very low	Low	Regular	High	Very high	Don't know/No answer
**24.1**	***What level of effectiveness (protects against disease) do you consider to have the influenza vaccine?***	1	2	3	4	5	6
**24.2**	***What level of safety (does not cause health problems) do you consider that you have the influenza vaccine?***	1	2	3	4	5	6
**24.3**	***What level of risk of contracting the disease do you consider you have?***	1	2	3	4	5	6
**24.4**	***What level of severity (can cause serious health problems) do you consider influenza to have?***	1	2	3	4	5	6

25. How much do you agree with the following statements about influenza

QUESTION		In full agreement	Partially in agreement	Undecid ed	Partially agree	Totally agree	Don't know/ No answer
**25.1**	***The influenza vaccine is very effective (protects against disease)***	1	2	3	4	5	6
**25.2**	***The influenza vaccine has side effects (harmful to health)***	1	2	3	4	5	6
**25.3**	***Influenza vaccine is hard to come by***	1	2	3	4	5	6
**25.4**	***Influenza Vaccine Causes Reactions***	1	2	3	4	5	6
**25.5**	***It is advisable to vaccinate against influenza every year***	1	2	3	4	5	6
**25.6**	***Only minors and the elderly should be vaccinated against influenza***	1	2	3	4	5	6

**26**	*In your opinion, how important is the influenza vaccine to prevent the disease?*	**Nothing important …………………………………………….…………….**	**1**	
		**Less important ……………….……………………….……….…………………**	**2**	
		**Undecided ………………………………………………….…………………….**	**3**	
		**Something important …………………………………………….……….**	**4**	
		**Very important ……………………………………………………..………..**	**5**	
		**Don't know/ no answer …………………………………………………**	**6**	
**27**	*What has your experience*	**Very bad ……..……………………………………………..……………………….**	**1**	

	been on the occasions when you have been vaccinated against influenza?	**Bad ………………………… ……………………………………**<br>**Neither good nor bad. ………………………………………………**<br>**Good …………… …………………………………… …………**<br>**Very good ……………………………………………………..**<br>**I have never been vaccinated ……………………………………**	**2**<br>**3**<br>**4**<br>**5**<br>**6**	
**28**	*Have you heard of cases that have had a bad experience with the influenza vaccine?*	**Yes ………………………………………………..……………………**<br>**No ……………………………………………..……………………..**	**1**<br>**2**	*→ 30*
**29**	*What relationship did that person have with you?*	**Family …………………………………………………………….**<br>**Known (neighbor, work, close friend, etc.) ………………………… ..**<br>**Indirectly (reference from another person, news, etc.) …… ..**<br>**Another situation ____**<br>**(Specify)**	**1**<br>**2**<br>**3**<br>**4**	
**30**	*How do you consider your state of health?*	**Very bad ……..…………………………………….………………….**<br>**Bad ………………………… ……………………………………**<br>**Neither good nor bad. ………………………………………………**<br>**Good …………… …………………………………… …………**<br>**Very good ……………………………………………………..**	**1**<br>**2**<br>**3**<br>**4**<br>**5**	
**31**	*Do you consider that the available influenza vaccine is effective (protects against disease)?*	**Yes ………..…….…………………………..……..……………..……**<br>**No ……….………………………………..…...……………..……..**<br>**NS/NC …..………………………….……………..…………..…….**	**1**<br>**2**<br>**3**	
**32**	*Do you think the available influenza vaccine is safe (does not cause health problems)?*	**Yes ………..…….…………………………..……..……………..……**<br>**No ……….………………………………..…...……………..……..**<br>**NS/NC …..………………………….……………..…………..…….**	**1**<br>**2**<br>**3**	
**33**	*Do you consider that the influenza vaccine is the best way to prevent this disease?*	**Yes ………………….…………………..……..…………….………**<br>**No ………………………………………..…...…………………..**<br>**NS/NC ..…….………………………….……………..………….……..**	**1**<br>**2**<br>**3**	
**34**	*Has a doctor diagnosed you with any of the following diseases?*<br><br>*Surveyor: you can mark more than one option, up to a maximum of three (those that the person considers most important)*	**Tuberculosis**<br>**Asthma**<br>**Chronic bronchitis or emphysema**<br>**Heart disease**<br>**Rheumatic fever**<br>**Diabetes (high blood sugar)**<br>**High blood pressure (hypertension)**<br>**Stroke (brain bleeding)**<br>**Arthritis**<br>**Gastritis or gastric ulcer**<br>**Colitis**<br>**Renal disease**<br>**HIV AIDS**<br>**Depression or anxiety**	**1**<br>**2**<br>**3**<br>**4**<br>**5**<br>**6**<br>**7**<br>**8**<br>**9**<br>**10**<br>**11**<br>**12**<br>**13**<br>**14**	

		**Tumors, cancer**<br>**Osteoarthritis**<br>**Another disease lasting more than 3 months**<br>**Specify** ______________________________	**15**<br>**16**<br>**17**	
**35**	***Do you think that influenza could have any risk of complication for you from having (mention diseases)?***	**Yes** ……………………………………<br>**No** ……………………………………<br>**NS/NC** ……………………………………	**1**<br>**2**<br>**3**	
**36**	***Do you think that the influenza vaccine could have any risk for you for having (mention diseases)?***	**Yes** ……………………………………<br>**No** ……………………………………<br>**NS/NC** ……………………………………	**1**<br>**2**<br>**3**	
PRACTICES AGAINST INFLUENZA				
**37**	***What do you do mainly to avoid getting sick with influenza?***	**Nothing**……………………………………<br>**I get vaccinated (specify where)** ______________________<br>______________________________<br>**I take care of myself (specify how)**______________________<br>______________________________<br>**I visit the doctor periodically to check me** ……… ..…. ………<br>**I am used to taking vitamins to protect myself** ……….. .. ………..<br>**I am used to taking food and / or herbal medicine that protect me**<br>**I am used to taking naturopathic medicine to protect myself** ……<br>**Other (specify)**______________________________<br>______________________________	**1**<br>**2**<br><br>**3**<br><br>**4**<br>**5**<br>**6**<br>**7**<br>**8**	 *→ 39*
**38**	***Have you ever been vaccinated against influenza? (check option 6 of question 27)***	**Yes** ……………………………………<br>**No** ……………………………………	**1**<br>**2**	 *→ 41*
**39**	***When was the last time you had a flu shot?***	**Less than a month ago** ……………………… ..… ..….<br>**Between 1 and 6 months** ……………………… .. ..<br>**Between 7 and 12 months (one year)** ………………………<br>**More than 1 year and up to 2 years** ……………………… .<br>**More than 2 years and up to 3 years** ………………. ……<br>**More than 3 years and up to 5 years** ………………. ……<br>**More than 5 years** ………………………………	**1**<br>**2**<br>**3**<br>**4**<br>**5**<br>**6**<br>**7**	
**40**	***What was the main reason you decided to get vaccinated?***	**To protect me from illness…. …………………. ... ………**<br>**On the recommendation of health personnel (doctor, nurse, social worker)** …… .. ……… .. ……………. ……………. ... ……<br>**For recommendations on the radio, TV, health campaigns, etc....**<br>**On the recommendation of a family member, friend, acquaintance, etc.** ……………………………………<br>**By indications in my work.** ……………………. ……… .. ……	**1**<br><br>**2**<br>**3**<br><br>**4**<br>**5**	*→ 42*<br><br>*→ 42*<br>*→ 42*<br>*→ 42*<br>*→ 42*<br>*→ 42*

		**Other: ____** **(Specify)**	**6**	
**41**	***What was the main reason you decided not to get vaccinated?***	**I didn't know it existed (check answer to question 13)… .. ……** **No one told me that I should be vaccinated …………………………….** **On the recommendation of a family member, friend, acquaintance, etc. ……** **I don't trust the vaccine …………………………………………… ..…** **Because it is harmful ……………………………………. ……… .…** **Other: ____** **(Specify)**	**1** **2** **3** **4** **5** **6**	
**42**	***On whose recommendation would you be vaccinated (or vaccinated) against influenza?***	**Health personnel (doctor, nurse, social worker)…. ……** **Recommendations on radio, TV, health campaigns, etc. …… ....** **Family, friend, acquaintance, etc.. …… .. ……………… .. …… ..…** **By indications in my work. ……………………. ……… .. ……** **Other: ____** **(Specify)**	**1** **2** **3** **4** **5**	

Interviewer: Thank the participation.
