# Supplementary material for: Influenza vaccination hesitancy in five countries of South America. Confidence, complacency and convenience as determinants of immunization rates
Source: PLoS One. 2020 Dec 11;15(12):e0243833. doi: 10.1371/journal.pone.0243833 (PMC7732123; doi:10.1371/journal.pone.0243833)
Supplement: Attachment — Submitted filename: Response to peer review PONE.pdf [file pone.0243833.s003.pdf]

October 2nd, 2020

Dear Holly Seale,

We thank very much the reviewers for their most helpful commentaries and suggestions to improve our work. We have responded to all comments as shown in the table below and look forward to any further comments

Response to peer review PONE-D-20-15450

**General comments**	**Reviewer #1**	The paper reports the rates of vaccine hesitancy in respect to the adult influenza vaccine across five countries within South America. The confidence, complacency and convenience framework is use to assess different aspects of vaccine hesitancy. These factors are compared across risk groups, countries and to self-reported influenza vaccine uptake.	We appreciate these comments.
	**Reviewer #2**	This manuscript will make an important contribution to the larger literature on influenza vaccine hestiancy, particularly as it covers immunization in an understudied region. However, I think a revision is necessary to strengthen the connections between the vaccine hestiancy framework, particularly the 3 C's, and the study's research questions and conclusions.	We appreciate these comments.
	**Reviewer #1**	The paper is well written and the topic is novel and importance to our understanding of global vaccine hesitancy. The data set clearly contains some important finding, however, the way in which you report the 3c factors and some of the statistical tests are confusing and would benefit from reworking.	We appreciate these comments.
**Introduction**	**Reviewer #2**	I would have liked to see **more explicit research questions**. There are a lot of different factors at play here -- multiple risk groups, many different countries, several different theoretical constructs, etc. Outlining research questions may help the reader focus on the significance of this work and keep both the results and discussion sections more focused.	We appreciate the comments and suggestions to focus on the research questions. We now propose three research questions and clarify the research objective.
		Given the title of the paper, I was expecting **more focus on the 3 C's**. I find this overview of the constructs to be a bit **superficial** and lacking in **support**. Please **incorporate more of the existing literature on vaccine hesitancy**. Given	We are thankful for this comment. We now provide a more in-depth overview of the 3C model based on the quantitative research reported in the literature for influenza vaccination hesitancy.

		that the conceptualizations of constructs at this stage is carried through to the development of survey measures and in analysis, this has significant bearing on the meaning of the results. Although a very different population, a recent study by Quinn et al. 2019 may be helpful. Quinn, S. C., Jamison, A. M., An, J., Hancock, G. R., & Freimuth, V. S. (2019). Measuring vaccine hesitancy, confidence, trust and flu vaccine uptake: Results of a national survey of White and African American adults. Vaccine, 37(9), 1168-1173.	
		Ln 68: What does "mandated" mean in this context? I associate this with mandatory or required vaccination, yet the following sentences seem to suggest this is not the case.	We substitued "mandated" with "recommended and publicily financed for"
		Ln 73: What would an ideal vaccination rate be? I was surprised to see rates this high.	We now cite WHO recommendations of reachibng desirable vaccination rates of 90% for 2020.
**Methods**	**Reviewer #1**	***1. Reporting of the 3c subscales:***	
		- As I understand it you have added the items of each of the 3c sub scales and then turned them into a number between 1 and 100 so that they are comparable. Then you have reported these as a percentage. I concerned that this makes your findings confusing, as when you report percentages it makes your finding sound like each component is a binary and the percentage is referring to how many people are e.g. complacent/not complacent (i.e. not a scale variable)<br>I suggest **change these to a number between 1 and 10**. This would make reporting clearer and distinguish these from where you report percentages of categorical variable (e.g. when you report vaccine uptake rates). If this change is made the analysis would need to be re-run, however, the conclusions would obviously remain the same.	We have rescaled the variables as suggested to report our results more clearly.
		Table 1 confuses the issue further. For the "Confidence in the vaccine" indicator you write "Resulting sum is rescaled to the interval (0,1) and expressed as %". If I was looking at this out of context, I would assume that you had split the sample into high and low confidence and were reporting the percentage of those in one of the categories.	Text in the table is now restated in terms of the new categorization.
		2. Comparison ANOVAs and Table 3:	

		The use of letters to signify significance makes this table difficult to understand. It also means that the reader cannot see the level of significance or the effect size. My advice would be to **check other papers in Plos One and see how they report multiple ANOVAs** like this.	We are maintaining the use of letters to represent significance as this device allows us to compare significant differences between pairs of countries. However, we now specify the alpha level in the table footer **to the level <.001**
		Type 1 errors: With the number of comparison ANOVA tests that you have conducted I am concerned with the **possibility of false positives** in your conclusions. At minimum a **note of caution** should be included in the discussion, however, I personally would **reduce the alpha level throughout**. This would of course change the conclusions of your analysis, especially for those results that are borderline significant, however, it would remove any spurious results.	We have reduced the alpha level to <.001. Most results were significant at this level, except when noted.
		As alluded to earlier, **more information** is needed **on item development** and any **reliability/validity tests** that were performed. Ln 133: Since the 3 C's are so central to the arguements of this paper, it is worth describing the development of measures in greater detail in the body of the text, not just in a table. Specifically, what each indicator was designed to measure and then how well it measures it.	We now report on the procedure used for the development of indicators and cite the relevant references.
	**Reviewer #2**	Also **more information on the choice to combine all respondents into a single "high-risk"** sample may be needed. **What differences exist between risk groups and between countries**? I need to be convinced that the measures are actually measuring what the authors claim they are.	Reflecting on the pertinence of combining all respondents ino a single group, we coma to the conclusion that it is not meaningful and have therefore deleted in tables and text reference to these aggregate values. We focus now the analysis on differences for each indicator across risk groups and countries.
		Ln 121: How does this **sampling approach** introduce **bias**? In what ways did the resulting sample differ from a more resprsentative sample?	We now discuss in the limitation of the study the possible biases of our sampling strategy with respect to service utilization.
**Results**	**Reviewer #2**	The **indicators scores need to be contextualized to be meaningful.** What does a convenience indicator of 74.8% mean in this context? I'm not sure what I'm supposed to take away from these results. I'm also not sure **what is a "good" or "acceptable"** level for any of these indicators and which are "bad" or "unacceptable" levels. **Starting with how these scores correlate to vaccine behavior may be more helpful.**	We have revised the interpretation of the 3C indicators to avoid comparison across them, and limit comparison within each indicator across risk groups and countries. However, we consider it reasonable to compare indicators across each other with respect to their association with vaccination status.

		ln 183: Why are **vaccination rates so much lower than those described in the introduction?** Any ideas?	We take up in the Discussion the differences between reported national vaccionation rates and our results.
		Ln 200: The differences in **vaccination rates between Chile and Uruguay are pretty extreme**. How does this impact the dataset? Did the 3 C's function the same across different countries?	As stated in the Introduction, countries were chosen across the continuum of vaccionation coverage. We discuss differenvrd in the 3C and reported vaccination rates across countries in lines 622 to 636.
**Discussion**	**Reviewer #2**	With so many results to touch upon, the **discussion section feels a little unfocused**. Again, tying the paper more closely to the vaccine hestiancy framework and following a clear set of research questions may help tighten the focus of the paper.	We have formulated three research questions and refocused the discussion accordingly.
		Some of these statments need to be further explained in much more detail. For instance, Ln 238-239. **What does it mean to suggest that convenience is better than confidence and both are better than complacency?**	Following from our response to reviewer 2' s comment for the Results section above, we now avoid comparing across the 3C indicators, except when discussing their association wih vaccination status. We have therefore removed the disciussion of comparisons across indicators.
		Ln 301 this paragraph feels more like a literature review than a part of the discussion. How is it tied to the study's findings?	We now integrate the two references in this paragrpah as similar evidence that is similar to our study on the importance of confidence and complacency.
**Minor comments:**	**Reviewer #1**	Line 105: **Cross-section (rather than transversal)** would be a more accessible, and the standard, term to use for this type of study.	We have changed the wording
		Line 119 -120: Please direct the reader to the section with the listed **ethics details** here.	We have directed the reader to the ethics details as suggested
		Line 146-147: Talking in terms of a higher and lower values and not “100 means less complacently” e.g. **a higher value on complacence variable indicates a lower level of complacence.**	We have modified the wording accordingly
		Line 169-172: As per the clarity of the 3c’s comment these would then be: **“the highest normalised score for convenience (7.48, out of a possible 10)” etc.**	We have modified the wording accordingly
		Line 173 - 182: Each comparison in this section should be accompanied with the reporting of a statistical test to demonstrate **significant differences**. Or **point towards a table that reports all comparisons.**	We are now stating that results are at the $p<.001$ unless otherwise stated. We are also pointing to table 3 for further details.

		Line 183 -185: This sentence would benefit from rephrasing. Something like **"…with children and older adults reporting significantly higher levels of vaccination than the other two risk groups",** then point to a table reporting the means and statistical test.	We have modified the wording following the suggestion.
		Line 202 -213: If these are correlations, please **report the correlation coefficients** for each. Either in the text or on the table.	No, these are not correlations. The analysis is based on analysis of variance models.
		Table 1:	
		Change "Points added gradually" to "**The scale was additive**"	We have modified the wording
		In "Complacency A. Influenza risk" construction you have the word "**Idem**.", it is unclear what this means.	We have modifided the wording, now stating "Same as above".
		Table 5:	
		**Report all p values without first "0", i.e. "<.01"**	We have modified deleting the 0.
		**A p< .001 level of detail would be useful here.**	We are now reporting at the <.001 level.
		Supplemental materials:	
		Some parts of the questionnaire in the supplemental materials still require translating.	We have completed the translation
		Data set should be made fully available	We are now making the dataset available.
